# Supplementary material for: Alternative Oxidase Alleviates Mitochondrial Oxidative Stress during Limited Nitrate Reduction in Arabidopsis thaliana
Source: Biomolecules. 2024 Aug 11;14(8):989. doi: 10.3390/biom14080989 (PMC11353033; doi:10.3390/biom14080989)
Supplement: Supplementary file 1 [file biomolecules-14-00989-s001.zip › SUP_FIGURES_Otomaru.pdf]

Supplementary Figure 1

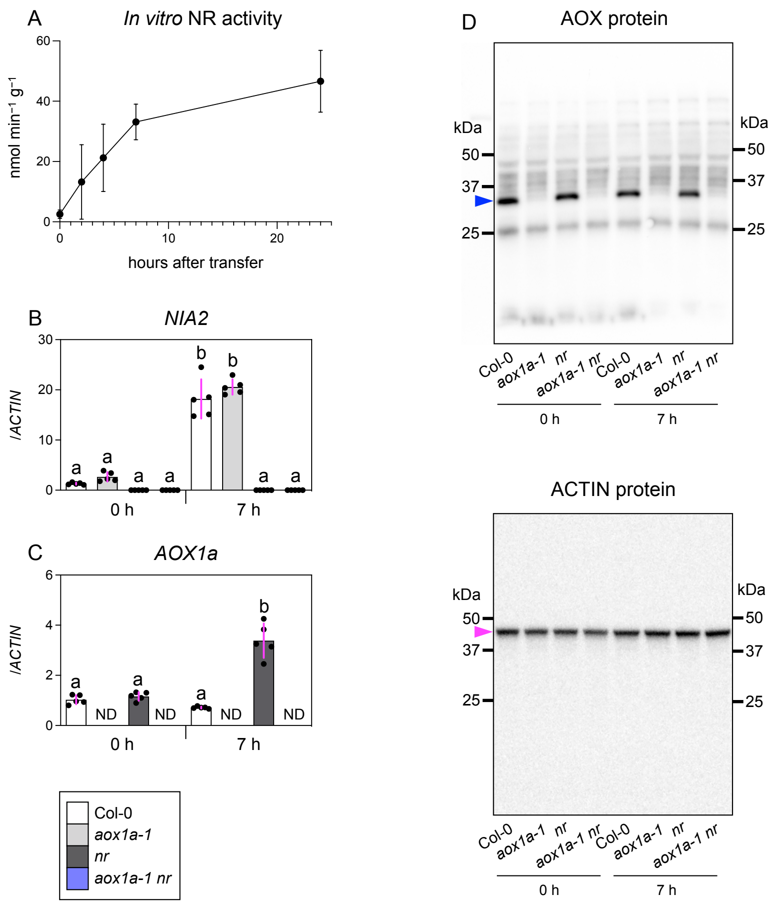

Fig. S1. Manipulation of activities of nitrate reduction and AOX.
(A) Time-course of *in vitro* NR activity in Col-0 shoots after nitrate supply. Data: mean ± SD (n = 6). Shoots from eight plants per plate were pooled as one biological replicate. (B,C) RT-qPCR analysis of *NIA2* (B) and *AOX1a* (C) in shoots before and 7 h after nitrate supply. Two plants of each line (eight in total) per plate were grown, and four shoots were pooled as one biological replicate. Data: mean ± SD (n = 5). Different lowercase letters indicate significant differences determined via Tukey–Kramer tests at $P < 0.05$. (D) Immunodetection of AOX and ACTIN isoproteins with specific antisera (Unclopped images). The blue and magenta arrowheads denote the signals corresponding to AOX and ACTIN, respectively. The contrast of images was linearly enhanced by using “enhance contrast (saturated pixels: 0.1%)” in Image J ver. 2.1.0/1.53c.

Supplementary Figure 2

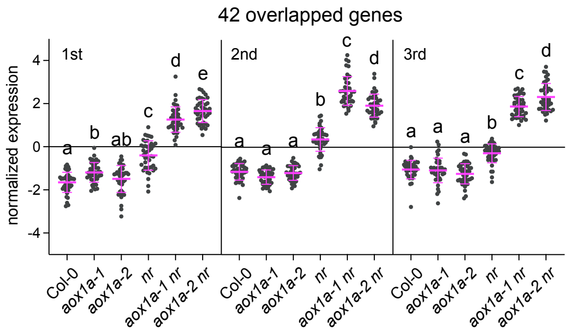

Fig. S2. Normalized expression of 42 overlapped genes.
Normalized transcript levels of the 42 genes are shown in Table S5. Different lowercase letters indicate significant differences determined via Tukey–Kramer tests at $P < 0.05$.

Supplementary Figure 3

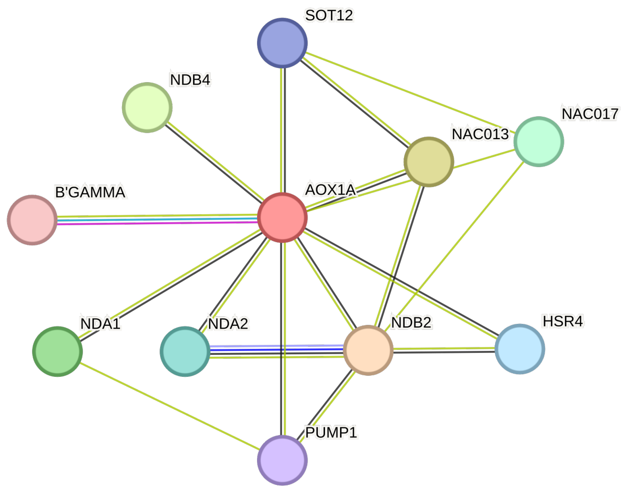

Fig. S3. Ourputs from STRING database ver 12.0. using AOX1a (AT3G22370) as query.
